# 

**Published:** 1916-08-12

**Authors:** 


### CONTENTS.

PAGE

**Our Hospitals and the National Relief Fund...** 441

**Children of the State: the Nation's Debt to its Warriors** ... ... ... ... ... 442

**Hospital and Institutional News** ... ... ... 443

A Red-Cross Kitchener Souvenir—City of London Lying-In Hospital: Appointment of Secretary—War Charities Registration Bill—The Tuberculous Soldier—The Detention of Time-Expired Doctors—A Small-Pox Hospital Project at Exeter—Books for Prisoners of War—The Surgical Tuberculosis Hospital at Newport—The Corner-Stone of a Hospital—The Future of Brunswick House—Another Extension at Coventry Hospital—The Scotch "Star and Garter"—The Store on the Terrace—The Welsh Medical School Delay—An Appeal to the Workers of Cardiff—Munificent Bequests to Glasgow Charities—Complaints at a Staffordshire Sanatorium—Two New Courses of Training for Women—Military Hospitals and Unnecessary Telegrams—Quakers' Relief Work in Russia—A Temporary Ward Burnt to the Ground—Hospital Attendants Killed in Action—Degradation in Monmouthshire—The Spiritualists' Ambulance—Substitutes for Cocaine—The Walker-Gordon Milk Laboratories—The August *Southern Cross*—This Week's Drug Market.

**Infantile Diphtheria** ... ... ... ... ... 449

A Special Danger in the First Year of Life.

**Relief in Belgium** ... ... ... ... ... ... 450

PAGE

**Our Hospitals and the National Relief Fund** 451

A Provincial Hospital Secretary's Views.

**The Representative Meeting of the British Medical Association** ... ... ... ... 452

**The Institutional Library** ... ... ... ... 453

The After-Treatment of Operations—More Minor Horrors—A Practical Book for Tuberculosis Officers: I.K. Therapy in Pulmonary Tuberculosis—Red Cross and Iron Cross—Under Three Flags: With the Red Cross in Belgium, France, and Serbia—Downward Paths—Surgical Nursing and Technique—The Campaign for Clean Milk—A Text-Book of Physics and Chemistry for Nurses—Surgical Bandages and Dressings.

**The Matrons' and Sisters' Department** ... 455

The Future of British Nursing.

Round the Hospitals.

The College of Nursing Register—The Need of Promptitude—Centralised Examinations—Poor-Law Masters' and Matrons' Association Faulty Statistics *re* Poor-Law Nurses—Special Quarters for Convalescent Nurses.

**Parliament and the Profession** ... ... ... 457

**Economy in the Isolation Hospital** ... ... 458

**The College of Nursing, Limited...** ... ... 459

**Brave and Gallant Medical Officers** ... ... 459

**The Middlesex Hospital Reports** ... ... ... 459

**Matrons' and Sisters' Appointments** ... ... 460

**Passing Events and Topics...** ... ... ... 460

**The Institutional Worker** ... ... (Suppl.) 1

